# Metabolomics based inferences to unravel phenolic compound diversity in cereals and its implications for human gut health

**DOI:** 10.1016/j.tifs.2022.06.011

**Published:** 2022-09

**Authors:** Rhowell Jr. N. Tiozon, Kristel June D. Sartagoda, Luster May N. Serrano, Alisdair R. Fernie, Nese Sreenivasulu

**Affiliations:** a Consumer Driven Grain Quality and Nutrition Unit, Rice Breeding and Innovation Platform, International Rice Research Institute, Los Baños, 4030, Philippines; b Max-Planck-Institute of Molecular Plant Physiology, Am Mühlenberg 1, 14476, Potsdam-Golm, Germany

**Keywords:** Cereals, Metabolomics, Phenolics, Gut microbiota

## Abstract

**Background:**

Whole grain cereals are a good source of nutrients. Several cutting-edge metabolomic platforms have been deployed to identify various phenolic compounds and enhance cereal bioactive bioavailability. A diet rich in cereal phenolics may modify the microbial composition, support gut homeostasis, and increase gut health, thereby lowering the risk of non-communicable illness.

**Scope and approach:**

In this work, we reviewed current metabolomic breakthroughs in cereal phenolic profiling and their effects on human health via gut microbiota modulation. We argue that the information presented in this paper will assist in the development of nutritionally superior cereal breeds and functional foods.

**Key findings and conclusion:**

Most cereal grains contain ferulic acid derivatives, caffeoyl glycerides, and feruloyl and coumaroyl esters. While there has been significant progress in discovering novel phenolic compounds in cereals, quantifying these molecules, and translating their therapeutic effects from animal model systems to humans remains a challenge. To this end, metabolomics, and other high-throughput-omics-based platforms must be integrated to further examine the structure and functionality of phenolic metabolites to breed nutritionally rich cereals as well as map their influence on human health benefits. Rare alleles must be introduced to improve bioactive content in cereal grains while maintaining yield. Following that, these exceptional varieties must be effectively processed to maximize phenolic bioavailability.

## Highlights

• Phenolic compounds are critical in avoiding metabolic disorders associated with oxidative stress.
• Breeding cereal crops to enrich phenolic compounds in grains contributes to personalized nutrition.
• A diet rich in cereal phenolics likely to increase human gut health, thereby lowering the risk of non-communicable illness.

## 1 Introduction

Cereals represent a significant source of calories and nutrients for the world's constantly rising population, especially in poorer nations. According to the Global Burden of Diseases, Injuries, and Risk Factors Study, dietary risk factors are responsible for around 8 million deaths per year (Vos et al., 2020). Epidemiological research has established that daily intake of whole-grain cereals reduces the risk of non-communicable illnesses (Swaminathan et al., 2021). Phenolic compounds are critical in avoiding disorders associated with oxidative stress. The phenolic compounds of cereal grains exist in both free and bound forms, and their contents vary depending on the genotype and environmental factors (Rao et al., 2018; Shao & Bao, 2015). The polyphenolic content of the cereal bran layer makes it a great source of antioxidants (Iftikhar et al., 2020; Sheflin et al., 2015). Consideration is given to the use of bioprocessing methods to increase the bioavailability of phenolic compounds in bran and cereal grains in order to create functional foods (Kasote et al., 2021).

In recent decades, the application of untargeted metabolomics in cereals (primarily in rice and wheat) has advanced considerably to quantify and profile wide array of metabolites. However, the wide array of metabolic diversity present in cereals makes it practically impossible for the metabolome to be determined using a single protocol (Alseekh, Bermudez, De Haro, Fernie, & Carrari, 2018). Nevertheless, combining GC-TOF-MS or GCxGC-TOF-MS and LC-QTOF-MS/MS metabolomics can decipher the entire spectrum of cereal metabolites and is well positioned to characterize the dynamic nature of phenolic chemicals under various conditions. In addition to monomeric anthocyanins, several dimalonylated monoglucosides of cyanidin, peonidin, and pelargonidin exist in pigmented cereals (Table 1). Since anthocyanins are the primary chromogenic chemicals found in colored grains, they have been the subject of a considerable number of targeted metabolomics (Abdel-Aal et al., 2016; Chen, Nagao, Itani, & Irifune, 2012; Hosseinian, Li, & Beta, 2008).

**Table 1:** Common flavonoids in whole-grain cereals measured using HPLC (mg/100 g DM).

	Barley	Corn	Oats	Rice	Rye	Sorghum	Wheat
**Anthocyanidins**							
Cyanidin	0.86–23.93(n = 7)	0.6–260.1 (n = 6)	npr	0–302.22 (n = 8)	0.29 (n = 1)	dominantly 3-deoxyanthocyanidins	0–7.1 (n = 13)
Maldivin	0.06–3.86 (n = 7)	nd		0–81.2 (n = 9)	nd		5.2–9.04 (n = 2)
Delphinidin	2.2–16.7(n = 7)	+		+	0.03–0.29 (n = 1)		0–4.5 (n = 13)
Peonidin	0.33–3.75(n = 7)	5.2–26.2 (n = 6)		0–11.87 (n = 8)	0–5.3 (n = 11)		0.27–0.6 (n = 2)
Pelargodinin	1.21–4.22(n = 7)	1.1–41.1 (n = 6)		+	nd		0–0.4 (n = 13)
**Flavan-3-ols**							
Catechin	1.31–2.38 (n = 7)	7.36 (n = 1)	0.56 ± 0.05 (n = 1)	0–1.39 (n = 11)	+	0–10 (n = 3)	0.83–1.79 (n = 75)
Epicatechin	0.18–0.50 (n = 4)	+	+	0.34–1.41 (n = 3)	+	0–2 (n = 3)	+
**Flavanones**							
Eriodictyol	+	nd	npr	+	npr	0–1.29 (n = 13)	npr
Hesperidin	0.18–0.56 (n = 4)	nd	npr	+	npr	+	3 × 10^−5^ – 0.02 (n = 100)
Naringin	0.0–0.1 (n = 7)	+	+	+	npr	npr	7 × 10^−5^ – 0.01 (n = 100)
Naringenin	0.0005–0.02 (n = 4)	14.8 (n = 1)	+	0–0.35 (n = 3)	+	0–4.84 (n = 13)	2.39–4.62 (n = 75)
**Flavones**							
Apigenin	+	+	npr	1.44–2.85 (n = 8)	0–1.52 (n = 12)	0–20.37 (n = 13)	20.0–36.5 (n = 21)
Isovitexin	0.190–0.89 (n = 4)	npr	+	0–1320 (n = 1)	npr	+	+
Luteolin	+	+	+	0.5–1.0 (n = 1)	+	0–18.2 (n = 13)	3.10–4.14 (n = 75)
Vitexin	21.82–93.57 (n = 4)	npr	+	1863–1965	npr	+	0.89–2.66 (n = 75)
**Flavonols**							
Quercetin	0.0004–18.41 (n = 4)	0.09–1.58 (n = 2)	10.18 ± 0.06 (n = 1)	0–1.87 (n = 11)	+	0–0.67 (n = 10)	1.96–10.48 (n = 75)
Myricetin	2.77–4.26 (n = 7)	+	+	0–0.4 (n = 3)	+	+	+
Rutin	0.02–1.12 (n = 4)	2.74–14.15 (n = 2)	0.32 ± 0.08 (n = 1)	0.24–0.38 (n = 3)	+	+	0.63–1.45 (n = 75)
Kaempferol	1.12–2.39 (n = 7)	0.124–224 (n = 2)	0.97 ± 0.2 (n = 1)	0–0.38 (n = 3)	+	0–0.48 (n = 10)	1.04–2.27 (n = 75)
*References*	(Ge et al., 2021; Kim et al., 2007)	(Méndez-Lagunas et al., 2020; Yang et al., 2019)	Bei et al., (2017)	(Chen et al., 2012; Huang & Ng, 2012; Kim et al., 2018)	Ravisankar et al., (2020)	(Dykes et al., 2009; Gu et al., 2008)	(Abdel-Aal et al., 2016; Hosseinian et al., 2008; Suchowilska et al., 2020)

+ = present but not quantified, nd = not detected, npr – no publish results.

**Fig. 1:**
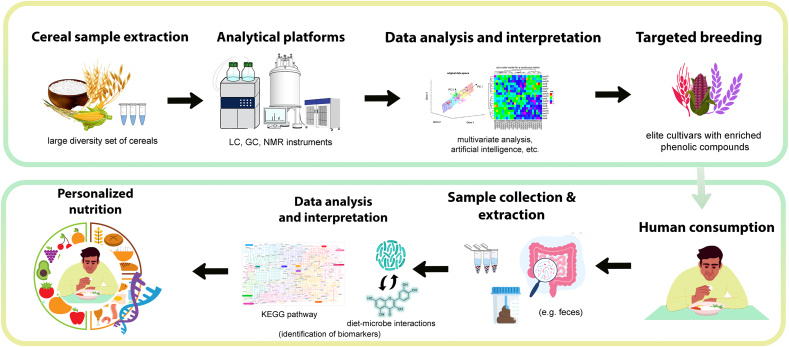
Metabolomic approach for targeted breeding and its effect in the gut microbiome. LC – Liquid Chromatography, GC – Gas Chromatography, NMR – Nuclear Magnetic Resonance.

Metabolomics, when combined with other high-throughput technologies like genome sequencing, transcriptomics, proteomics, and metagenomics, can reveal new information about the structure and function of phenolics in grains, as well as their impact on human health. Clearly, metabolomics coupled with genome-wide association studies (GWAS) to study metabolic variation in diversity panels, or quantitative trait loci (QTL) analysis of inbred lines permitted a more comprehensive understanding of the genetic basis of phenolic chemicals in cereals. Metabolomics is important not just for defining metabolites in grains, but it can also be used to find phenolic biomarkers in humans when deployed in clinical studies. Fig. 1 shows the workflow process on how the metabolomics approach in cereals can be utilized in breeding nutrition-dense cereals for gut well-being. Metabolomic and metagenomic analyses of the human gut revealed a mechanism by which cereal phenolics promote the growth of beneficial bacteria and the production of bioactive metabolites while inhibiting the proliferation of pathogenic microflora, all of which may influence human health and proclivity to develop certain diseases throughout life.

Some phenolic compounds have a high resistance to absorption in the small intestine due to their structural complexity. Hence besides tapping into the potential of enriching polyphenols in cereal grains comprehensive understanding of bioavailability and their biological activities in promoting gut health is critical. Next-generation sequencing deployed to profile the gut microbiome and metabolome characterization of metabolic intermediates have shown a link between the gut microbiota and almost every facet of human health through its influence on host immunological and metabolic responses (Catalkaya et al., 2020; Owolabi, Dat-arun, Takahashi Yupanqui, & Wichienchot, 2020). A cereal-phenolic-rich diet can induce a shift in certain key microbial species, promote gut homeostasis, and improve overall gut functional capacity (Catalkaya et al., 2020). This review will not extensively discuss the analytical platforms in cereal metabolomics since this has been accomplished elsewhere (Khakimov, Bak, & Engelsen, 2014). It will, however, detail the recent metabolomics advancements in cereal phenolics profiling and their modulatory effects on human health to prevent non-communicable diseases through the gut microbiota.

### 1.1 Advances in metabolomics approaches to unravel phenolic compound diversity in cereals and millets

**Fig. 2:**
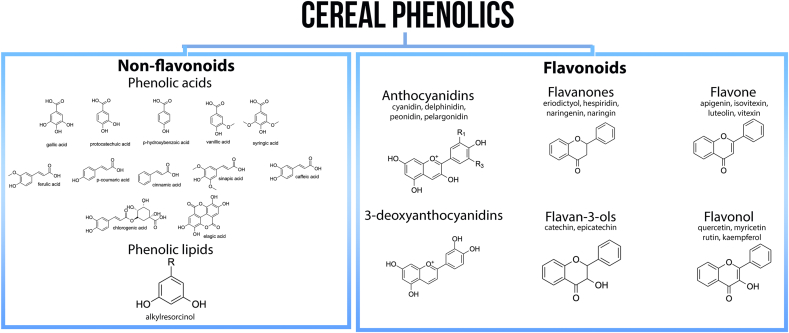
Cereal phenolic compounds quantified in targeted metabolomics.

Ferulic acid is the primary phenolic acid discovered at high amounts in all cereal grains (Supplementary Table 1). Most cereal grains' free and bound fractions contain abundant ferulic acid derivatives, caffeoyl glycerides, and feruloyl and coumaroyl esters. Fig. 2 enumerates the phenolic acids and flavonoids found in cereals. However, while there have been significant breakthroughs in detecting flavonoids in cereals (Table 1), gaps in quantifying these compounds remain. Furthermore, the metabolomics methods' extraction solvents and analysis tools had a significant impact on the final profile (Supplementary Table 2). It is noteworthy that the extraction parameters play a vital role in accurate phenolic compound measurement (Supplementary Table 2). For instance, the ultrasound-assisted extraction process obtained a higher phenolic and anthocyanin extraction yield than maceration and microwave-assisted extraction in corn and rye. The following discussion will highlight the recent advances in phenolic compounds inferred in major cereals and millets.

### 1.2 Barley

Barley is mainly used in malt and beer production but is known to contain wide array of phenolic compounds with superior nutritional value in human food (Khakimov, Jespersen, & Engelsen, 2014). Various chromatographic techniques, but primarily those coupled to mass spectrometric detection, have been employed to detect and quantify these phenolic compounds in barley seeds (Supplementary Table 1).

Metabolomic analysis of phenolic compounds has revealed that barley has high levels of benzoic acid-derived phenolics as compared to other cereals such as rye, wheat, and oats (Khakimov, Jespersen, & Engelsen, 2014). The phenolic acids in barley are present in the largest proportion in the bound form. Drawbridge, Apea-Bah, Hornung, and Beta (2021) reported high bioaccessibility ranging from 131 to 173% and 51–135% for ferulic and *p*-coumaric acids, respectively (Drawbridge et al., 2021). Ferulic and its precursor *p*-coumaric acids are the dominant phenolic acids detected across barley genotypes, regardless of the extraction parameters used (Supplementary Table 1). Hence, an increase in the level of *p*-coumaric is correlated to the promotion of biosynthesis of ferulic acid. This finding could explain why these two are among the most common phenolic compounds in cereals. Ge et al. (2021) identified 156 phenolic compounds using LC-MS-IT-TOF in four hulless barley grains with different colors. Black barley showed the highest phenolic acids, while white barley was detected with high flavonoid content (Ge et al., 2021). Kim et al. (2007) have classified the blue, black, and purple barleys based on pigmentation and implied that the darker the pigmentation, the higher the anthocyanin content (Kim et al., 2007). However, despite this finding, Quinke variety which grows on the Tibetan plain where it is highly exposed to UV-B, accumulates high levels of phenylpropanoids (Zeng et al., 2020).

In a metabolomics analysis coupled with the GWAS approach, the genetic regions responsible for the formation of phenolic acids were elucidated, and the enzyme agmatine coumaroyl transferase was determined to be responsible for the biosynthesis of phenolic amides in barley (Wiegmann et al., 2019). Besides genetic contribution, abiotic stress exposure greatly affects phenolic metabolites (Han et al., 2020). Barley has a higher tolerance to drought than other crops (Wiegmann et al., 2019). The significant accumulation of ferulic acid and sinapic acid in recombinant inbred barley lines under early and late stages of drought suggested a more robust accumulation of phenolic compounds under drought conditions (Piasecka et al., 2017). Generally, the most significant changes in biotic and abiotic stress were observed for ferulic acid, naringin, and acylated glycosides of flavones (Piasecka et al., 2017). Ferulic acid is one of the most effective photoprotectants that protect plants under drought conditions. Furthermore, 43 metabolites were remarkably altered by drought stress, most of which are flavonoids and their derivatives. These were then correlated to two hub genes belonging to the UDP-glycosyltransferase (UGT) family and three hub genes belonging to phenolic metabolism (Han et al., 2020). Further profiling and quantifying bioactive compounds in barley can elucidate more metabolites like *N*1,*N*8-dicaffeoyl spermidine, which were recently detected in barley (Drawbridge et al., 2021).

### 1.3 Corn

Corn is one of the most important staple crops extensively investigated for the added value of nutrition with enriched bioactive, vitamin A, and lysine content. Furthermore, corn has the most considerable level of bound ferulic and total phenolics compared to other cereals. Compared to regular corn, sweet corn has a greater proportion of free phenolics and flavonoids (Zhang et al., 2017). Still, a high abundance of glycosylated phenolics such as protocatechuic acid *O*-glucoside has been reported in three different cultivars of sweet corn kernels. Chinese sweet corn varieties contain ferulic and *p*-coumaric acids as the dominant phenolics (Zhang et al., 2017). Pigmented corn, such as blue and purple corns, contains phenolic acids such as caffeic acid, syringic acid, and anthocyanins which are not present in yellow corn (Méndez-Lagunas, Cruz-Gracida, Barriada-Bernal, & Rodríguez-Méndez, 2020). Other flavonoids such as eriodictyol, luteolin, isoorientin, and maysin are found in the pollen, silk, and tassel of corn but undetected or measured at low levels in seeds (Zhang, Liang, Yin, Li, & Wu, 2018). Some flavonoids in corn, such as flavanol–anthocyanins, have been identified but not yet quantified (Table 1).

The accumulation of phenolic acids such as gallic acid, chlorogenic acid, syringic acid, and hydroxycinnamic acid was attributed to increased maize phenylalanine-ammonia lyase (*ZmPAL*) expression at the beginning of corn cultivation. Meanwhile, the expression patterns of the maize chalcone synthase gene (*ZmCHS)* and maize anthocyanidin synthase (*ZmANS)* correspond to the accumulation of flavonoids and anthocyanin in corn, respectively (Zhang et al., 2020). Interestingly, flavonoids play a vital role in the evolutionary process of corn, where it is a crucial constituent of interspecific metabolic divergence. Genome-wide association study coupled with untargeted metabolomics of kernels from several corn inbred lines has revealed more than 28 flavones and the genes regulating their biosynthesis (Wen et al., 2014).

Further applications of corn metabolomics in breeding programs to understand the natural variation is undertaken to produce novel functional foods. Several studies have shown that the phenolic abundances differ among the cultivars and as well impacted by the environment (Frank, Röhlig, Davies, Barros, & Engel, 2012; Yang et al., 2019). Specifically, the results showed that the differences in the metabolomic profiles of genetically and non-genetically modified corn grown in two locations (Germany and South Africa) were related to environmental variability rather than genetic modifications. Multivariate data assessment further revealed that environmental factors such as growing locations and seasons were dominant parameters that drive variability of the metabolite profiles in corn (Frank et al., 2012). The wide range of metabolic variation noted in diversity panels unraveled through metabolomics analysis and linking with GWAS unraveled key candidate genes for the novel metabolites in the formation of phenolic acid and hydroxycinnamic acids (Wen et al., 2014). The gene locus *CCoAOMT1* (GRMZM2G127948) was found responsible for the content of *N*-(caffeoyl-O-hexoside)-spermidine (S8) and two of *N, N*-caffeoyl,feruloyl-spermidine derivatives. The acyl receptor, *N*-hydrocinnamoyltransferases that use aliphatic amines, and donor hydroxycinnamoyl-CoA were the key enzymes identified in the biosynthesis of phenolamides. GWAS indicated that the locus *PHT* (GRMZM2G030436) was highly associated with the metabolite diferuloylputrescine (DFP) (Wen et al., 2014). DFP is the predominant phenolic amide in the corn kernel, although its physiological role is still unknown, while others report that DFP plays a role in radical scavenging.

Purple corn grown at two locations (lowland and highland) showed primary and secondary metabolite composition differences. Lowland varieties are susceptible to high temperature, moisture, and increased concentration of pests calling for extensive use of pesticides (Ranilla et al., 2021). Therefore, some are grown at Andean altitudes above sea levels so that there would be a lower frequency of pests during cultivation in organic conditions (Ranilla et al., 2021). Further research is needed to understand the metabolic differences in purple corn adapted to the environmental conditions of the Andean region.

### 1.4 Oats

Oats (*Avena sativa* L) have higher levels of certain phytochemicals and can endure more harsh growth conditions (for example, wet climates and acidic soils) than other cereals. Soycan et al. (2019) quantified 22 commercial oats and revealed the highest total phenolic acids in oat bran concentrate > oat bran > flaked oats > rolled oats > oatcakes (Soycan et al., 2019). Most phenolic compounds are present in the bound form, where ferulic, sinapic, and caffeic acids are present at the highest levels (Soycan et al., 2019).

Generally, the adsorption capacities of phenolic acids are lowered by the methylation and methoxylation in the oat β-glucan. The increase in adsorption capacities follows the order flavonol > flavone > flavanone > isoflavone (Soycan et al., 2019). Oats contain anthocyanin levels ranging from 5 to 90.6 mg/100g DM (Table 1). Through the application of solid-state fermentation, free catechin and bound ferulic acid content increased by 100-fold (Bei, Liu, Wang, Chen, & Wu, 2017). Similarly, when oats germinate, a considerable rise in ferulic and *p*-coumaric acids is observed.

Avenanthramides, classified as phenolic alkaloids, are amides of various cinnamic acids with different anthranilic acids. They are unique to oats, with around 25 avenanthramides being recorded to be present. They are primarily present in the outer grain layer with their levels increasing during maturation and ranging from 394.8 to 1518.6 mg/kg FW (Table 1). The avenanthramide content in oats is negatively influenced by high nitrogen fertilization. Still, it does not vary between conventional and organic cropping systems as supported by UHPLC-PDA-MS-based metabolomics in oat grain (Allwood et al., 2019). By contrast, low molecular weight phenolics increased in elevated nitrogen fertilization (Allwood et al., 2019). Chen et al. (2018) demonstrated a considerable variation in the phenolic and avenanthramide contents, although it should be noted that all tested varieties exhibited anticancer properties (Chen et al., 2018). Wild oat accessions were differentiated from cultivated oats by their fatty acid and sugar content (Loskutov et al., 2017). Cultivated oat was reported to have increased oleic acid but decreased linoleic acid content (Loskutov et al., 2017). Future research may include a more comprehensive targeted approach to quantify phenolic compounds in oat grains, infer their interaction with other metabolites, and identify their response to various stresses, including fungal infections, submergence, and oxidative stress.

### 1.5 Rice

Among the phenolic acids, ferulic acid is the most abundant in the endosperm, bran, and whole-grain rice, while *p*-hydroxybenzaldehyde and *p*-coumaric acid are predominant in the husk (Huang & Ng, 2012). The concentration of phenolic compounds is preferentially enriched in bran and embryo compared to endosperm (Shao & Bao, 2015). Distinct phenolic fingerprints are found in traditional rice varieties. Among the six unique phenolics, cirsimaritin has demonstrated strong antihyperglycemic potential (Haldipur & Srividya, 2021). Chinese wild rice has shown to display higher flavonoid content than non-pigmented rice, which is attributed to enrichment in wild rice's phenylpropanoid biosynthesis pathway (Yu et al., 2021). Furthermore, wild rice species were reported to contain proanthocyanidins with (+)-catechin, (−)-epicatechin, and (−)-epigallocatechin present in both terminal and extension units (Yu et al., 2021).

Kim et al. (2021) have demonstrated the use of six analytical instruments to elucidate the overall metabolome patterns in different types of rice and present the complex interactions between primary and secondary metabolites (Kim et al., 2021). A significant variation in flavonoids is found between pigmented and non-pigmented rice (Huang & Ng, 2012). LC-MS/MS-based targeted metabolomics revealed 90 flavonoids in rice. The UHPLC-QqQ-MS-based metabolomics approach identified 159 flavonoids, among which 78 showed differential abundance (Yu et al., 2021). The metabolic variation of diversity panels in indica was unraveled through mGWAS analysis, which identified superior haplotypes for bHLH transcription factor among red rice samples with enriched catechin and low glycemic index property (Brotman et al., 2021). Through the combination of metabolomic techniques, phenolic acids such as methyl *p*-hydroxycinnamate, 8–5′ decarboxylation dimer of ferulic acid, and 5-5′/8′-O-4″ dehydrotriferulic acid, and flavonoids such as isoorientin, isoorientin 2″-O-glucoside, and vitexin 2″-O-glucoside were identified in rice (Kim et al., 2018). It seems highly likely that the integration of metabolomics with other -omics approaches can unravel unique phenolic compounds in rice and provide new insights into the phenolic compound mechanism that can be translated to various health benefits. A final example that is worthy of note in rice is the identification of apigenin 5-*O*-glucoside as conferring potent UV-B tolerance (Peng et al., 2017). However, whether the chemical attributes of this metabolite possess bioactive property remains to be assessed.

Untargeted metabolomics in rice subjected to bioprocesses such as fermentation and germination increased the total phenolic content and induced new phenolic derived metabolites (Kasote et al., 2021). Both fermentation by *Rhizopus oryzae* (AS3.866) and extrusion increased free, bound, and total phenolics in defatted rice bran to varying degrees (Chen et al., 2019). Cooking can significantly alter the phenolic composition of rice, specifically the degradation of anthocyanins (Tiozon, Sartagoda, Fernie, & Sreenivasulu, 2021). Multivariate analysis of glycosylated flavonoids and hydrolyzed tannins discriminated between cooked black and red rice (Rocchetti et al., 2021). Nevertheless, changes in the phenolic profile did not attenuate the anti-inflammatory activities of black rice (Tiozon et al., 2021).

### 1.6 Rye

Most phenolic acids in rye are found in the bound form, with the free form accounting for only 1–5% of total phenolic acids. The majority of phenolic acids seen in the bound form are esterified, while some are linked with lignins (Supplementary Table 1). This profile may be a result of cultivation techniques. For instance, organically grown rye contains higher ferulic and benzoic acid than rye grown via conventional contemporary means (Mishra, Sarkar, Zwinger, & Shetty, 2017). The concentration of phenolic acids in rye milling fractions increased in the order rye bread < rye flour < rye grain < rye bran. The soluble fraction of rye endosperm flour contains a higher amount of ferulic acid and a lower level of sinapic acid, whereas, an opposite trend was quantified in the insoluble-bound fraction of rye endosperm bread (Koistinen et al., 2018). While processing methods such as sourdough fermentation reduced lignans marginally, they enhanced other metabolites such as branch chain amino acids and microbial metabolites derived from phenolic acids (Koistinen et al., 2018).

Ferulic dehydrodimers such as 8,5′-diferulic acid, 5,5′-diferulic acid, 8-O-4′-diferulic acid, and 8,5′-diferulic acid benzofuran form were quantified with the total concentration of 27.8–40.9 mg/100g DM. The bound phenolics present in the insoluble dietary fiber (556.6 ± 8.9 mg GAE/100 g DM) are higher than in the bran (417.4 ± 6.1 mg GAE/100 g DM) of rye (Supplementary Table 1). However, a high amount of bound phenolics are not efficiently metabolized by the human body (Iftikhar et al., 2020). Furthermore, the presence of phenolic acids affects the sensory attributes of rye. In particular, vanillic, veratric, and syringic acids, and other lignans such as pinoresinol, potentially render a perceived aftertaste of bitterness rye-based products.

The combination of HPLC-DAD and HPLC-QTOF MS has provided insight into the structural diversity of flavonoids in the rye. Flavonoids that were detected as *C*-glycosides are orientin, vitexin, vicenin 2, schaftoside, luteolin *C*-pentoside *C*-hexoside, chrysoeriol *C*-hexoside, chrysoeriol *C*-hexoside *C*-hexoside, apigenin *C*-pentoside *C*-pentoside, and apigenin *C*-hexoside-*O*-hexoside (Pihlava, Hellström, Kurtelius, & Mattila, 2018). That said, rye flavones are predominantly detected in *O*-glycosides present as derivatives of chrysoeriol and tricin (Ravisankar, Queiroz, & Awika, 2020). Zykin, Andreeva, Lykholay, Tsvetkova, and Voylokov (2018) estimated that cyanidin and peonidin are the main anthocyanin aglycones found in various colors of rye. The purple grain has the highest anthocyanin content (Zykin et al., 2018). On the other hand, Lykholay, Vladimirov, Andreeva, Smirnov, and Voylokov (2014) discovered rye mutants possessing non-allelic genes without anthocyanins (Lykholay et al., 2014). Besides phenolic acids and flavonoids, rye contains diverse phenolic lipids and alkylresorcinols in 72–148 mg/100 g (Pihlava et al., 2018). Saturated alkylkresorcionls such as 15:0, 17:0, 19:0, 21:0, 23:0, and 25:0 were isolated from rye grains (Hammerschick, Wagner, & Vetter, 2021). Besides alkylresorcinol's dietary benefits, such as antioxidant and DNA protective effects, it can be used as a nutritional biomarker (Kaur, Sandhu, Purewal, Kaur, & Singh, 2021). Furthermore, rye contains an abundant source of lignans, mostly syringaresinol derivatives. To describe the diversity of phenolic compounds in rye more thoroughly, comprehensive fingerprinting and quantification of the many discovered flavonoids and anthocyanins is required.

### 1.7 Sorghum

Sorghum is considered a functional food as grains possess a wide array of bioactive compounds, namely phenolic acids, flavonoids, phytosterols, and polyphenols. Several studies have used metabolite information in breeding high-yield sorghum intending to improve the yield and quality of the crop (Alseekh et al., 2018). Sorghum kernels exist in diverse colors, and these differences are correlated to the significant variation in their antioxidant activities and phenolic contents (de Morais Cardoso, Pinheiro, Martino, & Pinheiro-Sant'Ana, 2017). Another benefit of the consumption of pigmented sorghum is the strong correlation between flavonoid abundance and decreased starch digestibility (Rocchetti et al., 2020). Thus, pigmented sorghum has the dual advantage of having a greater antioxidant potential and a decreased caloric content. The bioactive phenolic compounds isolated from sorghum subjected to *in vivo* and *in vitro* analyses have shown the potential to elicit beneficial changes in populations with non-communicable diseases such as obesity, diabetes, and cardiovascular diseases (de Morais Cardoso et al., 2017).

Dykes and Rooney (2006) have reviewed and enumerated the flavonoids and proanthocyanidins in sorghum. However, this compilation mostly drew from old records, and the level of these compounds was not stated (Dykes & Rooney, 2006). The proanthocyanidins (also known as tannins) in sorghum are mainly composed of catechins as the main chain terminating units. Although, the presence of prodelphinidins, proluteolinidin, and proapigeninidin have also been reported (Reeves et al., 2020).

Generally, black sorghum contains the highest level of 3-deoxyanthocyanidins, yellow sorghum has abundant flavanones, and tan sorghum is rich in flavones (Dykes et al., 2009, 2011). The dominant flavones in sorghum are luteolin and apigenin, where the former is present in glycoside form and the latter in aglycone form (Dykes et al., 2011). In a few black and brown sorghum accessions, condensed tannins such as catechins, epicatechins, epigallocatechins, and epiafzelechin were detected from 217 differentially expressed metabolites (Zhou et al., 2020). Moreover, 43 metabolites were commonly detected among white, red, and purple sorghum seeds, and a high level of cyanidin *O*-malonyl-malonyl hexoside, cyanidin *O*-acetylhexoside, and cyanidin 3-O-glucosyl-malonylglucoside were detected at a significant level in the three cultivars (Zhou et al., 2020). The sorghum's most common and unique anthocyanin type is the 3-deoxyanthocyanidins, including the orange luteolinidin and yellow apigeninidin. These compounds lack a hydroxyl group at the C-3 position, making them more stable to pH-induced color change than the anthocyanins(Gu, House, Rooney, & Prior, 2008). The use of UHPLC-ESI-QTOF-MS/MS was able to identify 110 phenolic compounds in sorghum, 56 of which were recently identified as new flavonoids such as glycitein, formononetin, ononin, and hispidulin (Ofosu et al., 2021). Another untargeted metabolite profiling study utilizing the GC-MS platform identified 221 metabolites, which varied considerably among the 61 cultivars studied under tropical and temperate conditions. Clustering analysis revealed that the sorghum grain metabolomes possess distinct metabolic diversity to adapt to their environments. In general, those grown in temperate climates showed high levels of phenylpropanoids (Ramalingam et al., 2021).

Sorghum with brown pericarp contains the highest proanthocyanidin levels, whereas lower levels were identified in black pericarp sorghum (Rao et al., 2018). GWAS conducted using multi-locus models from *S. bicolor × S. halepense* recombinant inbred lines identified 55 quantified trait loci (QTLs) with a hotspot covering 19 putative glutathione *S-*transferase genes associated to anthocyanins and as well identified *MYB-bHLH-WD40* complex genes linked to flavonoid and proanthocyanidin biosynthesis in sorghum (Habyarimana, Dall’Agata, De Franceschi, & Baloch, 2019). Four genomic selection models (GBLUP, BRR, BL, and BayesB) were used to test their ability to predict the genetic merit of 114 sorghum genotypes for the production of polyphenols, flavonoids, and condensed tannins. For polyphenols and flavonoids, the prediction accuracy ranged from 0.53 to 0.56, total antioxidant capacity from 0.49 to 0.52, and condensed tannins from 0.55 to 0.58 (Habyarimana et al., 2019).

### 1.8 Wheat

Cultivated wheat (*Triticum* aestivum) composes of diploid, tetraploid, and hexaploid species. Among these, the hexaploidy bread wheat has been grown globally and thus has been subject to the most extensive metabolomic studies. Several studies have confirmed that the genotypes of *Triticum* species (e.g., spelt, einkorn, emmer, bread wheat, and durum wheat) can be differentiated based on the long-chain saturated substation of the alkylresorcinol (Rascio et al., 2016). Furthermore, the differences in 17:0 to 21:0 alkylresorcinol homolog ratios have been employed to detect and quantify the adulteration in whole grain durum flour and dried pasta (Rascio et al., 2016).

Suchowilska, Bieńkowska, Stuper-Szablewska, and Wiwart (2020) compared four wheat species of 75 breeding lines containing a wide range of phenolic acids and flavonoids, in which the bran layer constitutes the abundant concentration. Certain species of wheat, particularly *T. turanicum* have higher flavonoid concentrations than other wheat species, albeit with no difference in antioxidant capacity (Suchowilska et al., 2020). In comparing ancient and modern wheat varieties, ancient wheat varieties do not only have a greater diversity of phenolic compounds like ferulic acid isomers (Boukid et al., 2019); but also contain higher phenolics, flavonoid, and alkylresorcinol content (Boukid et al., 2019). Ma et al. (2016) reported nine phenolic acid biosynthesis pathway genes that exhibited three distinct expression patterns during grain filling. Among the types of wheat (e.g., white, purple, and red), white wheat had higher total phenolic content in the early stages of seed development. In comparison, purple wheat had higher total phenolic content in the later stage of seed maturation (Ma et al., 2016). Concurrently, 237 phenolic compounds were identified in wheat's early and mature stages, with greater concentration in the early stage (Santos et al., 2019). The study of wheat represented the first attempt to characterize the effect of domestication on the metabolome (Beleggia et al., 2016). This study revealed that reduction in unsaturated fatty acids was associated with selection during the domestication of emmer (primary domestication). We also show that changes in the amino acid content due to selection mark the domestication of durum wheat (secondary domestication).

Coupled with genetic analysis, metabolomics has brought new insights into the flavonoid structural and genetic diversity, which was utilized to elucidate the wheat's flavonoid decoration pathway (Khlestkina, Shoeva, & Gordeeva, 2015). Chen et al. (2020) recently identified 26 flavonoid-related candidate genes with confirmed enzymatic actions (e.g., glucosylation and subsequent malonylation of various flavonoids). In addition, *TaMYB10 and TaMYC1* genes were also reported to play a vital role in anthocyanin synthesis by regulating key genes linked with the flavonoid pathway (Wang et al., 2020). These genes are overexpressed in pigmented wheat varieties rendering higher flavonoid concentrations than in non-pigmented varieties. Among colored wheat types, purple wheat contained a greater concentration of anthocyanins and was identified with 13 different anthocyanins (Hosseinian et al., 2008). Further investigations into the flavonoid-related alleles may aid us in comprehending regulatory networks of anthocyanin biosynthesis in different wheat types.

### 1.9 Millet

Millets, also referred to as coarse cereals. The most important species are browntop, finger, little, foxtail, proso, kodo, pearl, and barnyard millet, containing phenolic chemicals with distinct identities and levels. Generally, ferulic and *p*-coumaric acids are present in soluble and bound fractions of the different millet cultivars. The bound fractions contain higher levels of these phenolic compounds than the soluble ones and exhibit high antioxidant activities (Bartwal, Pande, Sharma, & Arora, 2016). Among the small-seeded millets, finger millets are extensively farmed in India and Africa and are thus the most researched millet species. Compared to rye, oats, and barley; finger millet has superior nutritional value due to its greater calcium, dietary fiber, phytates, proteins, and mineral content. Finger millet is notable for its enhanced content of bound phenolic compounds such as 8-8′-aryltetralin-DFA, 5-5′-DFA, 8-5′-DFA benzofuran, and 8-*O*-4′-DFA which are dihydrodimers of ferulic acid. Moreover, the genetic potential of finger millet to endure abiotic stress such as drought is noteworthy (Bartwal et al., 2016). The *PRM6107* and *PR202* finger millet genotypes were determined to have the highest stress tolerance by activating ROS scavenging antioxidative enzymes (Bartwal et al., 2016). A comparison of the phenolic compounds in pearl and finger millets revealed that pearl millet contains less catechin and epicatechin, while finger millets contained only kaempferol glycoside. Moreover, procyanidins B1 and B2, as well as protocatechuic and *p*-hydroxybenzoic acids were only detected in finger millets (Hassan, Sebola, & Mabelebele, 2020).

Kodo millet showed a higher total free and bound phenolic content when compared to proso millet. A targeted mass spectrometry-based analysis revealed that the metabolomic profiles of four proso millet types differed, with colored proso millet grains having greater levels of ferulic, isoferulic, and syringic acid than white grains. In addition to hydroxycinnamic and hydroxybenzoic acid derivatives, isomers of chlorogenic acid, such as neochlorogenic and cryptochlorogenic, were found in all cultivars investigated in varying levels. Yadav et al. (2021) found that 16 genes have functional properties that are correlated to an antioxidant biochemical pathway, including the gene that encodes for isoflavone 2′-monooxygenase, which was then associated with the pathways involving carbamoyltransferase and UDP-glucosyl transferase (UGTs) (Yadav et al., 2021). This finding suggests that these genes may play a major role in promoting the formation of antioxidant-related flavonoid molecules through diverse chemical processes. Similarly, fifteen genes encoding UDP-glucuronosyl and UGTs are involved in starch metabolism, anthocyanin, and flavonol metabolism (Yadav et al., 2021).

A comprehensive untargeted and targeted profiling of metabolites among the four Zhangzagu progenies and their parental lines demonstrated a regulated accumulation of 300 metabolites belonging to various classes. The phenolic acid derivatives detected were *N*-feruloyl agmatine, *N*-feruloyl spermidine, *N′*, *N″*-disinapoylspermidine, *N*-hexosyl-*p*-coumaroyl putrescine, *N*-*p*-coumaroylputrescine *iso1*, and *N-*feruloyl putrescine (Li et al., 2018). Compared to genomic and transcriptomics-based research, millet grain metabolomics under biotic and abiotic stressors is still understudied. Thus, future directions in millet metabolomic investigations could be explored to complement present genomic discoveries, to get a better understanding of the complex biological processes occurring in wide array of millets for unravelling potential antioxidant molecules carrying human health benefits but also to assist in predicting its phenotype plasticity for climate change adaptation.

### 1.10 Gut-microbiota and human health implications of cereal-derived phenolic compounds

**Fig. 3:**
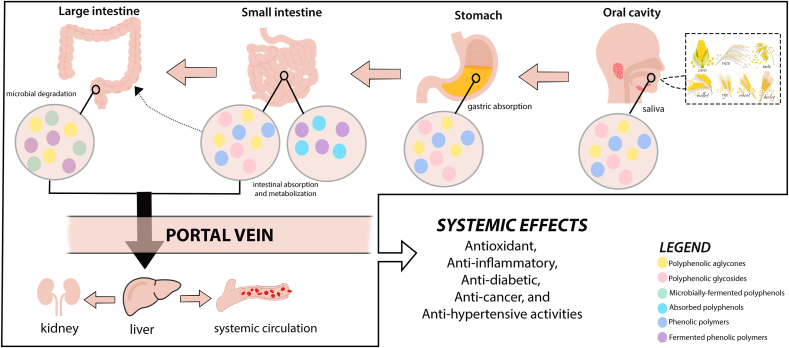
Unravelling the metabolism of cereal-derived phenolic compounds in the human body and its implication to nutrition.

Phenolic compounds and metabolites derived from cereal grains have been shown to have a wide range of beneficial impacts on human health (Fig. 3) (Tiozon et al., 2021). Recent advances in next-generation gene sequencing of stool, oral, and plasma samples established gut metagenome with human health. Systematic analytics of microbiome features project how these favorable biological properties are potentially mediated in part by gut microbiota metabolism via a reciprocal system of reliance inside the host to maintain human health (Catalkaya et al., 2020; Danneskiold-Samsøe et al., 2019). Similarly, these findings underscore the significance of gut homeostasis dysregulation with the onset of several significant clinical conditions, including allergies, metabolic syndrome, cancer, and other non-communicable diseases (Hasan & Yang, 2019). More recent studies reveal a relationship between gut microbiota dysbiosis and neurodevelopmental disorders such as autism spectrum disorder (Yang et al., 2016). Thus, nutritionally focused interventions toward positively influencing gut microbiota patterns may be a viable strategy for improving human health and well-being. One caveat to deciphering these intricacies is that the reciprocity between food, host, and microbiota is exceedingly personal, necessitating modern analytical approaches and bioinformatics to analyze big data to infer personalized nutrition aimed at optimizing human health (Danneskiold-Samsøe et al., 2019). To this end, several high-throughput omics-based methodologies have been employed alone or combined with conventional analytical techniques (Calderón-Pérez et al., 2020; Ramakrishna, Sarkar, Dogramaci, & Shetty, 2021). This section briefly examines the proposed mechanisms through which cereal phenolics regulate the gut microbiota and countering disease-related consequences. Additionally, deploying different omics-based approaches for deciphering the microbial repertoire and chemical alterations in the gut in response to cereal phenolics consumption is discussed (Table 2).

**Table 2:** Gut microbiota-modulating effects of cereal-derived phenolic compounds.

Biological Source	Phenolic compound/s of interest	Effects on microbiota	Method used for the study	Quantification of Phenolic Compounds	Reference
Barley	Protocatechuic acid, gallic acid, cinnamic acid, catechin, and dihydroxybenzoic acid	↓*Helicobacter pylori* *↑* LAB	*In vitro* incubation of *H. pylori* using special peptone agar and plate count method for LAB	RP-HPLC-DAD	Ramakrishna et al., (2021)
Corn	Anthocyanins, p-coumaric, ferulic, and caffeic acid	*↑* LAB	*In vitro* incubation using pure LAB cultures	UPLC-DAD	(Gálvez Ranilla et al., 2017)
Millet	3-hydroxybenzylhydrazine, luteolin-3′,7-diglucoside, N-acetyltyramine, p-coumaric acid, vanillin, sinapic acid, ferulic acid and isophoro	*↑Oscillospira, Ruminococcus* ↓*Allobaculum*	*In vivo* study using murine cecal samples; 16S rRNA gene sequencing to determine fecal microbiota	LC-MS	Liu et al., (2021)
Oat	Avenanthramide, hydroxycinnamic acids and benzoic acid derivatives	↑*Bifidobacterium,* Enterobacteriaceae*, Hafnia alvei, Bifidobacterium adolescentis*	*In vitro* batch culture fermentation using human fecal inocula; 16S rRNA gene sequencing to determine fecal microbiota	HPLC-DAD	Kristek et al., (2019)
Rice	Indole-2-carboxylic acid, Hydrocinnamic acid, Benzoic acid, Phenylacetic acid	↑*Methanobrevibacter, Paraprevotella, Ruminococcus, Dialister, Anaerostipes, Barnesiella, Bifidobacterium, Clostridium*	*In vivo* study using human fecal samples; 16S rRNA gene sequencing to determine fecal microbiota	GC-MS	Sheflin et al., (2015)
Rice	Lignans, Isoflavonoids, Phenolic alcohols, Isoflavones, Flavan-3-ols	↑*Clostridiaceae*, *Ruminococcaceae, Lachnospiraceae, Streptococcaceae*	*In vitro* large intestinal fermentation using pig fecal inocula; PCR characterization of bacterial profile	UHPLC-ESI/QTOF-MS	Rocchetti et al., (2019)
Rice	Pyrocatechol, 3-(4-hydroxyphenyl) propionic acid, salicylic acid, *trans*-cinnamic acid	↑*Bifidobacterium, Lactobacillus* ↓*Clostridium, Enterobacter, Bacteroides*	*In vitro* batch culture fermentation using human fecal inocula; FISH technique to enumerate microbiota	LC-MS	Owolabi et al., (2020)
Sorghum	3-deoxyanthocyanins	↑*Bifidobacterium, Lactobacillus, Roseburia, Prevotella*	*In vitro* batch culture fermentation using human fecal inocula; PCR characterization of bacterial profile	HPLC-DAD	Ashley et al., (2019)
Wheat	Dihydroferulic acid and ferulic acid	↓*Bifidobacteriales, Clostridium, Dialister, Blautia, Collinsella* ↑*Firmicutes, Bacteroidetes, Lactobacillus, Prevotella*	*In vivo* study using human fecal samples; 16S rRNA gene sequencing to determine fecal microbiota	HPLC-MS/MS	Vitaglione et al., (2015)

**Abbreviations**: Fluorescent in situ hybridization (FISH); GC-MS (Gas Chromatography–Mass Spectrometry); LAB (Lactic Acid Bacteria); LC-MS (Liquid Chromatography – Mass Spectrometry); RP-HPLC-DAD (Reversed-Phase High-Performance Liquid Chromatographic); UHPLC-ESI/QTOF-MS (Ultra-High-Pressure Liquid Chromatography Coupled to a Hybrid Quadrupole-Time-Of-Flight Mass Spectrometer); UHPLC-MS^n^ (Ultra-High Performance Liquid Chromatography – Mass Spectrometry).

The human gastrointestinal milieu, generally referred to as the gut, is populated by a plethora of commensal microbial species, predominantly bacteria. It is estimated that the number of bacteria living inside humans is 1.3 times more than the number of human cells (Catalkaya et al., 2020; Sarkar & Shetty, 2018). Owing to the richness of this ecosystem, the gut is regarded to be a unique and essential organ with functions that transcend beyond simple digestion. The heterogeneity of the human gut starts during birth, with the mode of delivery largely defining early microbial colonization. The newborn gut microbiota is expected to be profoundly affected by the nutrient density of diet (Yang et al., 2016). Although the microbiota may attain a reasonably stable condition upon maturity, food, age, antibiotic exposure, and other variables may impact the composition, diversity, and hence the ecological balance, of the gut microbiota throughout life (Hasan & Yang, 2019).

The health-promoting benefits of cereal phenolics are dependent on their bioaccessibility and bioavailability. Hence, various technological approaches have been developed to enhance the bioavailability (Kasote et al., 2021). It has been shown by *in vitro* digestion and colonic fermentation studies that germination, fermentation, nanoencapsulation, ultra-fine grinding, and some forms of heat treatment, among others, increase the extractability and bioaccessibility of phenolic components from cereals (Gabaza, Shumoy, Muchuweti, Vandamme, & Raes, 2016; Hithamani & Srinivasan, 2017; Kasote et al., 2021), although these observations vary depending on type of processing procedure applied (Kumari, Chandrasekara, & Shahidi, 2019). Pressure cooking, open pan boiling, and microwave heating of finger millet typically resulted in lower levels of bioaccessible phenolic compounds (30–35% less) than native samples, whereas, sprouting and acidification enhanced these levels by 20 and 10%, respectively (Hithamani & Srinivasan, 2014, 2017). Relative to its native form, extruded and fermented defatted rice bran samples had greater bioaccessible phenolic contents of 41% and 64.4%, respectively (Chen et al., 2019). The authors ascribed these results to the high amounts of bound phenolic components inherent to rice bran and alterations in the microstructure of the bran as a consequence of the fermentation and extrusion process. Evidently, unless cereals have been subjected to these processes, only about 5%–10% of their phenolic compounds are absorbed in the small intestine lumen upon ingestion, while the great majority (90–95%) reach the distal gut, where they undergo further biotransformation (Loo, Howell, Chan, Zhang, & Ng, 2020).

In the gut, there are two distinct but related mechanisms that have been proposed for how cereal phenolics, in their bound form, may benefit human health - by supplying prebiotics that aid in maintaining the gut's ecological homeostasis and by stimulating the formation of bioactive microbial metabolites (Catalkaya et al., 2020; Sarkar & Shetty, 2018). Prebiotic-like properties of dietary phenolic compounds have been shown to discriminately influence the composition and functional capability of the gut microbiota by supporting the development of helpful bacterial taxa while repressing the growth of harmful ones (Rocchetti et al., 2019). Fermented barley flour with high protocatechuic acid, gallic acid, cinnamic acid, catechin, and dihydroxybenzoic acid concentrations reportedly maintained the development of *Lactobacillus* while suppressing *Helicobacter pylori*, the bacterium which causes peptic ulcer disease in humans (Ramakrishna et al., 2021). In purple rice, pyrocatechol, 3-(4-hydroxyphenyl) propionic acid, salicylic acid, *and trans*-cinnamic acid were correlated to the increase in *Bifidobacterium* and *Lactobacillus* and substantial reduction in *Clostridium/Enterobacter* and *Bacteroides* populations (Owolabi et al., 2020). Furthermore, metagenomic characterization of the microbiota in feces collected from seven healthy people revealed that meals and snacks containing rice bran promoted the growth of *Bifidobacterium*, a bacterial species whose population dominates a healthy gut (Sheflin et al., 2015).

Consequent to these modulatory properties, short-chain fatty acids (SCFAs) and phenolic metabolites are produced in greater amounts. The gut, seen as a “bioreactor”, converts food-derived metabolizable phenolic phytochemicals into SCFA, primarily acetate, propionate, and butyrate (Owolabi et al., 2020). Apart from providing energy to colonocytes, regulating intestinal hormones (de Sousa et al., 2019), and participating in gluconeogenesis, SCFAs have been shown to have significant mitigating properties against a variety of diseases, including diarrhea, hypertension (Calderón-Pérez et al., 2020), ulcerative colitis, inflammatory bowel disease, Alzheimer's disease, low-grade and systemic inflammation (Roager et al., 2019). Another recognized health metric is the ratio of phyla *Bacteroidetes* to *Firmicutes*, often used as a biomarker for gut dysbiosis and as a hallmark of obesity and hyperlipidemia (Zhai et al., 2021). According to two separate research done in Japan (Kasai et al., 2015) and Ukraine (Koliada et al., 2017), obese individuals showed significantly higher *Firmicutes* levels and lowered *Bacteroidetes* levels than normal-weight and lean individuals. Using 16S rRNA sequencing, it was revealed that mice treated with anthocyanin from black rice had a 72% lower *Firmicutes/Bacteroidetes* ratio than mice on a high-fat and cholesterol diet (Wang et al., 2020). Interestingly, the makeup of the gut microbiota and the quantity of SCFAs reacted dose-dependently to black rice anthocyanin intervention. Additionally, the intervention appeared to control cholesterol metabolism and ameliorate gut microbiota dysbiosis in mice (Wang et al., 2020). Nutritional therapies focusing on cereals tend to have various effects when tested on human subjects. For example, in an eight-week diet trial conducted by Vitaglione et al. (2015), significant increases in *Bacteroidetes* abundance and decreases in the population of obesity-associated bacteria were observed, despite the absence of significant effects on anthropometric measurements and body composition (Vitaglione et al., 2015). On the other hand, Roager et al. (2019) discovered a significant decrease in body weight and only a slight increase in urinary whole grain-derived microbial metabolites, with no significant changes in fecal microbiota following eight weeks of multi-whole-grain cereal product consumption composed of oats, rye, and wheat (Roager et al., 2019). These findings were attributed to the distinct impact of different cereal grains on specific gut microflora. Further research is needed to fully comprehend the modulatory effects of certain grains on the human gut flora. In the context of systemic and low-grade inflammation and its link to an imbalanced gut microbiota repertoire, dietary interventions with cereal phenolics revealed a concomitant decrease in metabolic and inflammatory disease markers. For instance, a marked reduction in tumor necrosis factor-a (TNF-a), a rise in interleukin (IL)-10, and a tendency toward decreased plasma plasminogen activator inhibitor 1 were seen following whole-grain wheat intake (Vitaglione et al., 2015). Similarly, subjects supplemented with a high-rye diet and elevated circulating alkylresorcinol levels presented substantial reductions in interleukin (IL)-6 and C-reactive protein levels (Roager et al., 2019). Taken together, these results undoubtedly demonstrate the interconnectedness of gut microbiota to human health and diseases.

Concomitant to SCFA production, native phenolic compounds that resist digestion in the upper gastrointestinal tract reach the colon, where the gut microbiota catabolizes them through multiple enzymatic activities (Loo et al., 2020; Ozdal et al., 2016). Here, cereal phenolics, primarily phenolic acids such as hydroxycinnamates, hydroxybenzoic acids, and ferulic acids trapped within the cereal fiber matrix or linked through ester bonds, are converted into low molecular weight components by numerous gut bacterial species (Tiozon et al., 2021). The catalysis of a wide array of phenolics by gut microbiota, as well as the profiling of this vast array of low molecular weight metabolites must be systematically outlined through various metabolomics platforms to infer gut health. Moreover, this data needs to be associated with electronic health data and linked to occurrence of diseases in human subjects. For instance, *Lactobacillus* synthesize esterases that biotransform ferulic acid to 3-(31,41-dihydroxyphenyl) propionic acid, 31,41-dihydroxyphenyl acetic acid, 3-phenylpropionic acid, and benzoic acid (Ozdal et al., 2016; Vitaglione et al., 2015). Untargeted metabolic profiling using UHPLC-QTOF mass spectrometry of rice flour-based gluten-free cookies subjected to *in vitro* large intestine fermentation with pig fecal inocula revealed that the phenolic profile of the samples significantly changed during the assay. Using the comprehensive database on polyphenols, Phenol-Explorer, 14 phenolic metabolites, contributed significantly to the reported findings, which were linked back to the degradation of lignan isoflavonoids, phenolic alcohols, isoflavones, and flavan-3-ols (Rocchetti et al., 2019). Moreover, via GC-MS analysis of stool samples, it was shown that indole-2-carboxylic acid, phenylacetic, and benzoic acids, all of which are metabolites of ferulic and other hydroxycinnamic acids, were considerably elevated after 28 days of rice bran supplementation (Sheflin et al., 2015). Dihydroferulic acid was the most prevalent circulating metabolite in overweight/obese subjects after a diet trial of whole-grain rice (Vitaglione et al., 2015). Furthermore, increased *Lactobacillus* abundance was associated with decreased TNF-a, indicating another possible relationship between cereal phenolics and inflammation amelioration. It is clear that the capacity of the gut to biotransform polyphenols that have greater biological activity than their precursor structures cannot be discounted.

In a world where breakthroughs in nutrition research are increasingly driven by the predisposition of individuals to acquire certain diet-related diseases more often than others, personalized nutrition has lately attracted tremendous interest as a conceptual shift toward prolonging human life by preventing the onset or progression of chronic diseases (Tebani & Bekri, 2019). The premise of this innovation conveys the focus on customizing dietary choices for individual needs by utilizing phenotypic variables and genetic data. Although the proposition that individual dietary needs are contingent to anthropometrics, physiological states, biological and other environmental elements, personalized nutrition is an emerging field of study which will likely to take central stage to link nutrient density of diets to optimize human health (Chaudhary et al., 2021; Laddu & Hauser, 2019). The number of research that positions cereals within the context of personalized nutrition is limited and warrant special attention. We anticipate that with recent advances in next-generation sequencing and analytics, the scientific knowledge of cereal phenolics' role in human health and disease would expand what is presently understood of personalized nutrition. There are currently few studies evaluating and quantifying the prebiotic index of diverse phenolic substrates obtained from cereal grains. Additionally, it is crucial to explore the degree to which phenotypic diversity and technological approaches aimed at increasing cereal phenolic absorption affect that makeup the composition and function of the gut microbiota. Therefore, large-scale human clinical data are required to demonstrate the therapeutic effects of cereal phenolics as a dietary intervention in innovative nutritional approaches for treating gastrointestinal and systemic disorders.

## 2 Conclusion

The vast majority of the world's population relies on cereal production as a critical food supply. Cereals are a vital source of calories and contain secondary metabolites like phenolic compounds associated with a reduced risk of chronic diseases, particularly when whole grain consumption is promoted. Pigmented cereals possess a wide array of phenolic acids, flavonoids, and anthocyanins, which in particular contribute to the health of our gut microbiota. Several *in vivo* and *in vitro* studies have shown that phenolics in the human gastrointestinal milieu tract can increase good bacteria to maintain human health. Furthermore, it has multiple benefits in improving immunity against diseases and preventing different neurodevelopmental disorders. To demonstrate cereals' dietary benefits, we need to deploy various state-of-the-art metabolomics techniques (a) to determine the diversity and function of phenolic metabolites in cereals, (b) introduce the rare alleles to enrich bioactive in cereal grains without compromising yield, (c) deploy processing technologies to increase the bioavailability of these phenolics and (d) conduct metagenome-sequencing and metabolomics analysis to fingerprint gut microbiota-derived metabolites to map it to the genetic fingerprint of digital human health for bringing in personalized nutrition solutions.

This review presents important perspectives on the significance of metabolomics as a powerful approach for identifying metabolites in cereals and millets associated with various physiological conditions. Existing untargeted metabolite profiling studies provide a comprehensive approach to looking at phenolic compounds that have beneficial effects on human health. Moreover, an evaluation of the metabolite profile that aids in examining the variations in the phenotype based on genetic and environmental factors proves that metabolomics is a valuable tool in establishing a good selection criterion for desirable traits, including optimum nutritional content.

Since the metabolites detected using different analytical strategies vary depending on the extraction parameters and instrument used, it is important to develop optimized and validated protocols for different sample material to generate reproducible and reliable metabolomic data. Although there are significant breakthroughs in determining novel phenolic compounds, there are still missing gaps in quantifying these compounds. For instance, the anthocyanins in oats and sorghum have not yet been quantified, and flavonoids in other cereals. In this case, a relatively more significant number of samples should be analyzed to investigate the cereal metabolome properly. Screening promising cereal cultivars for the phenolic content and antioxidant capacity may help assist in breeding for more superior nutritional value. Furthermore, up scaling the targeted quantification assays using the multiple reaction monitoring modes and untargeted metabolic studies is pivotal in understanding the entire metabolome of each cereal.

Although marker-assisted selection and genomic selection technologies have revolutionized breeding programs to bring the missing nutrition into cereals, deploying single-cell metabolomics as part of clinical studies to study impact of cereal metabolomes and their potential human health benefits is warranted. Furthermore, machine learning can potentially generate novel information to link holistic solutions in the arena of personalized nutrition. Collectively, these advances will help accelerate the development of more comprehensive cereal metabolome solutions that could optimize crops to address food security, human nutrition, and disease prevention.

## Funding

NS acknowledges funding support from the RICE CGIAR Research Program, the UK Biotechnology and Biological Sciences Research Council UK Research & Innovation program (Project BB/T008873/1), the Agricultural and Processed Food Products Export Development Authority (APEDA), the Department of Agriculture and Farmers welfare, Government of India. This research was funded by the Academy for International Agricultural Research (ACINAR). ACINAR, commissioned by the German Federal Ministry for Economic Cooperation and Development (BMZ), is being carried out by ATSAF e.V. on behalf of the Deutsche Gesellschaft für Internationale Zusammenarbeit (GIZ) GmbH.
