# Supplementary material for: Metabolomics based inferences to unravel phenolic compound diversity in cereals and its implications for human gut health
Source: Trends Food Sci Technol. 2022 Sep;127:14–25. doi: 10.1016/j.tifs.2022.06.011 (PMC9449372; doi:10.1016/j.tifs.2022.06.011)
Supplement: Multimedia component 1 [file mmc1.docx]

Supplementary Table 1. The main phenolic acid profile of whole-grain cereals (in mg/100 g DM)

| No. of cereal samples | Extractant | TPC* | Instrument used for profiling | Caffeic acid | Chlorogenic acid | Cinnamic acid | Ellagic acid | Ferulic acid | Gallic acid | Protocatechuic acid | p-coumaric acid | p-Hydroxybenzoic acid | Sinapic acid | Syringic acid | Vanillic acid | Ref |
| --- | --- | --- | --- | --- | --- | --- | --- | --- | --- | --- | --- | --- | --- | --- | --- | --- |
| Barley | | | | | | | | | | | | | | | | |
| n=4 | 80% MeOH | 27.45-98.34 | LC-MS-IT-TOF | 0.005 – 0.06 | 0.22 – 1.69 | - | - | 0.007 – 0.54 | 0.002 – 0.01 | 0.003 – 0.014 | 0.004 – 0.24 | 0.001 – 0.15 | - | - | - | (Ge, et al., 2021) |
| n=4 | 80% MeOH | 85.37 -233.19 | HPLC-RP | 0.10-0.48 | - | - | - | 0.54-0.59 | - | - | 0.52-0.66 | - | 0.095-0.61 | - | 0.45-0.77 | (Drawbridge, Apea-Bah, Hornung, & Beta, 2021) |
| n=4 | 80% acetone | 384.6 - 415.0 | HPLC-PAD | - | 0.97-1.96 | - | - | 50.16-54.65 | 0.47-1.38 | 1.53-4.17 | - | - | - | - | - | (Deng, et al., 2020) |
|  |  |  |  |  |  |  |  |  |  |  |  |  |  |  |  |  |
| n=4 | 80% acetone | 333.9 - 460.8 | HPLC | 2.40 – 3.75 | 23.92-48.55 | - | - | 48.14 – 67.53 | - | 0.28 – 9.86 | 0.78 – 1.59 | - | - | - | - | (Zhu, et al., 2015) |
| n=20 | 85% acidified MeOH | 192.9-291.7 | UHPLC | 0.21-0.65 | 0.01-0.59 | nd | - | 0.03-0.31 | 0.05-2.05 | - | nd | 0.11-0.27 | - | 0.01-2.25 | - | (Suriano, et al., 2018) |
| n=10 | MeOH | - | ﻿HPLC-DAD-ESI-MS/MS | 0.08 – 0.39 | - | - | - | 0.01-0.43 | nd | nd | 0.01-0.21 | - | - | - | 0.03-0.14 | (Carvalho, Curto, & Guido, 2015) |
| n=10 | EtOH & NaOH | - | HPLC-DAD | nd | - | - | - | 22.83 – 41.26 | - | - | 0.53-6.65 | 0.72 – 2.9 | 2.61-3.51 | 0.43-1.48 | 0.17-4.09 | (Andersson, et al., 2008) |
|  |  |  |  |  |  |  |  |  |  |  |  |  |  |  |  |  |
| Corn/Maize | | | | | | | | | | | | | | | | |
| n=1 (blue corn) | MetOH | 141.92 ± 5.2 | HPLC | 2.74± 0.01 | 15.9± 0.01 | - | - | 82.27± 0.07 | - | - | 33.31± 0.0 | 2.39± 0.0 | - | 4.07 ± 0.0 | 8.15± 0.0 | (Méndez-Lagunas, Cruz-Gracida, Barriada-Bernal, & Rodríguez-Méndez, 2020) |
| n=1 | MetOH | - | HPLC-DAD | - | 4.84 ± 1.95 | - | - | 37.05 ± 4.82 | 9.79 ± 2.93 | - | 16.46± 1.98 | - | - | - | - | (Ramírez-Jiménez, Rangel-Hernández, Morales-Sánchez, Loarca-Piña, & Gaytán-Martínez, 2019) |
| n=1 (YT28) | 80% acetone | 909.5-920.3 | RP-HPLC | - | - | - | - | 1.16 | 0.109 | - | 0.933 | - | - | - | - | (Yang, et al., 2019) |
| n=4 (colored) | Acidified MetOH | 23-388 | HPLC | - | - | - | - | 0.41 -1.83 | - | 0.01-0.24 | 0.01-0.05 | - | - | - | 0.05 – 0.53 | (Hu & Xu, 2011) |
| Oats | | | | | | | | | | | | | | | | |
| n=22 (commercial) | 80% MetOH | 35.48-151.86 | HPLC-PDA | 1.66 – 8.15 | - | - | - | 21.41-1118.64 | - | - | 0.51-5.57 | 0.51-2.26 | 3.57-11.65 | 1.18 – 2.84 | 0.69-20.9 | (Soycan, et al., 2019) |
| n=1 | Acidfied 80% MetOH |  | HPLC | 0.05 ± 0.04 | 1.11 ± 0.02 | - | - | 12.72 ± 0.13 | 0.37 ±  0.01 | - | 0.94 ± 0.12 | 0.45 ± 0.04 | 1.57 ± 0.09 | - | 0.23 ±0.00 | (Bei, Chen, Liu, Zhang, & Wu, 2018) |
| n=8 (husked) | 80% MetOH | 132.1-168.7 | UPLC-MS | - | - | 0.72 – 1.21 | - | 53.30 – 74.77 | - | - | 59.0-82.6 | 0.47-0.67 | - | 0.39 – 1.08 | 0.46 – 0.71 | (Multari, et al., 2018) |
| n=1 | 80% MetOH and NaOH | 31 | HPLC | 0.54 ± 0.1 | nd | - | - | 0.65 ± 0.03 | 0.71 ± 0.00 | - | 0.43 ± 0.26 | 0.29 ± 0.03 | 2.91 ± 0.2 | - | 0.49 ± 0.07 | (Bei, Liu, Wang, Chen, & Wu, 2017) |
| n=1 | 80% acetone | - | RP-HPLC | 1.56 ± 0.08 | 1.93 ± 0.02 | - | - | 19.27 ± 0.55 | - | - | 2.08 ± 0.19 | 0.4 ± 0.05 | 0.71 ± 0.08 | 0.63 ± 0.0 | 0.73 ± 0.002 | (Zeng, Liu, Luo, Chen, & Gong, 2016) |
| Rice | | | | | | | | | | | | | | | | |
| n=18 (mix) | 80% MetOH | 39 - 209 | HPC-UV | - | - | - | - | 17.39-43.87 | 0.06-3.43 | 0.15-10.89 | 2.0-9.43 | 0.10-0.45 | 0.22-1.37 | 0.08-0.17 | 0.1-10.53 | (Pang, et al., 2018) |
| n=3 | Acidified 80% MetOH &NaOH | 40.6-136.6 | LC-MS/MS | - | - | - | - | 8.9-20.64 | - | 0.15-5.21 | 2.22-3.08 | 0 – 4.48 | 0.97-0.98 | 0.67-1.31 | 0.81-4.87 | (Shao, Xu, Sun, Bao, & Beta, 2014) |
| n=3 (mix: pigmented & non-pigmented) | Acidified acetonitrile | - | HPLC-DAD | 0.19 – 0.29 | - | 0.27-0.31 | - | 0.79-1.39 | 0.21-0.26 | 2.3-3.54 | 0.37-0.54 | 0.23-0.34 | nd | 0.21-0.38 | 0.30-2.48 | (Irakli, Samanidou, Biliaderis, & Papadoyannis, 2012) |
| n=11 | 80% MetOH | 24-178 | RP-HPLC | 0-3.16 | 0-0.2 | nd | 0-1.3 | 0-1.51 | nd | - | 0-0.34 | 0-5.99 | - | - | 0-3.23 | (Huang & Ng, 2012) |
| n=9 (mix) | 70% acidified MetOH & NaOH | 4.60-60.60 | RP-HPLC | 0.15-1.86 | - | - | - | 0.50-5.27 | 0.19-2.03 | 0.02-2.56 | 0.07-7.69 | 0.02-0.88 | - | 0.12-3.12 | 0.04-1.37 | (Vichapong, Sookserm, Srijesdaruk, Swatsitang, & Srijaranai, 2010) |
| Rye | | | | | | | | | | | | | | | | |
| n=19 | 80% MetOH | 98.4-336.9 | HPLC | - | - | - | - | 0.19-0.62 | - | - | 0.05-0.13 | - | - | - | 0.08-0.34 | (Kulichová, et al., 2019) |
| n=1 (cltvr Amilo) | Acetone- MetOH - water (2:2:1 v/v/v) | - | HPLC-DAD | 1.21 | - | - | - | 105.35 | - | - | 6.09 | 0.8 | 13.89 | 0.28 | 1.23 | (Pihlava, et al., 2015) |
| n=1 (cltvr Amilo) | Acetone- MetOH - water (2:2:1 v/v/v) | 65.3 | HPLC | nd | - | - | - | 0.6 | - | - | - | 0.3 | 0.2 | nd | 0.3 | (Heiniö, et al., 2008) |
| n=17 | NaOH | - | HPLC-RP | - | - | - | - | 94.1-117.4 | - | - | 3.7-6.5 | - | 9.3-14.4 | - | - | (Andreasen, Christensen, Meyer, & Hansen, 2000) |
| Sorghum | | | | | | | | | | | | | | | | |
| n=2 | - | 197.7 | HPLC | - | - | 0.08 | - | 4.31 | 0.02 | 0.84 | - | 1.11 | - | 1.57 | 0.54 | (A. P. T. Miafo, Koubala, Kansci, & Muralikrishna, 2020) |
| n=6 | MetOH | 68.44-580.40 | HPLC | - | - | 0.05-1.07 | - | 0-56.31 | - | 0-2.53 | 0-13.64 | 0-7.73 | 0-10.21 | 0-7.42 | 0.14-7.15 | (A.-P. T. Miafo, Koubala, Kansci, & Muralikrishna, 2019) |
| n=4 | MetOH | - | HPLC | 2.56-5.21 | - | 0.4-1.97 | - | 10.46-34.26 | 1.32-4.6 | 2.25-14.13 | 8.6-23.21 | 1.54-3.38 | - | - | 0.87-5.07 | (Dykes & Rooney, 2006) |
| Wheat | | | | | | | | | | | | | | | | |
| n=8 (whole wheat) | 80% acetone | 21.12 – 139.33 | RP-HPLC | - | - | - | - | 1.4-2.9 | - | - | 0-1.02 | 0.34-0.88 | - | 1.18-2.04 | 0.64-0.96 | (Tian, Chen, Tilley, & Li, 2021) |
| n=15 (durum wheat) | Acidified 80% MetOH | 177.48-272.62 | UHPLC-MS | 0-2.88 | - | - | - | 7.59-69.65 | - | - | 3.51-5.66 | - | 0-4.31 | - | - | (Boukid, et al., 2019) |
| n=12 | 80% EtOH | - | HPLC-UV | - | - | - | - | 45.86-86.23 | - | - | 1.43-2.71 | 0.5-1.13 | 4.49-7.46 | 1.07-2.07 | 0.87-2.24 | (Gotti, et al., 2018) |
| n=3 (including colored) | Acidified MetOH | 114.7-152.5 | HPLC | 0.01-0.03 | - | - | - | 56.39-83.87 | - | - | 1.30-2.85 | - | - | 0.8-0.96 | 1.67-2.84 | (Ma, et al., 2016) |
| n=37 | 80% EtOH | - | HPLC | 2.1-16.4 | 0-9.6 | - | - | 23.5-75.2 | - | - | 5.1-14.5 | - | - | 0-1 | 0-9.9 | (Zhang, Wang, Yao, Yan, & He, 2012) |

*assay using Folin-Ciocalteu method (mg phenolic acid equivalents/100g DM/FW)

- = not reported, nd = not detected

Supplementary Table 2. Application of metabolomic approaches to characterize phenolic compounds in cereal grains and other important findings

| Samples & description | Extraction solvent | Metabolomic  Approach | Analytical instrument | Phenolic metabolites | Key findings related to phenolic compounds | Ref |
| --- | --- | --- | --- | --- | --- | --- |
| Barley |  |  |  |  |  |  |
| n = 2 (XZ25 (low) and XZ20 (high)) | methanol: acetonitrile: water = 2:2:1 | Untargeted | UHPLC-QTOF-MS | 114 phenolic compounds were successfully identified and quantified: phenolic acids (28), flavanones (7), flavones (17), isoflavones (6), flavonols (20), anthocyanins (20), flavanols (11) and procyanidins (5) | There are 118 phenolic metabolites affected by drought stress. A weighted gene co-expression network analysis (WGCNA) of 17,424 highly expressed genes unraveled black (two hub genes belonged to UGT family) and turquoise modules (three hub genes belonged to phenolics pathway) that are significantly associated with phenolic variation. | (Han, et al., 2020) |
| n=8 | 80% methanol | Targeted | UPLC-PDA | phenolic acids, flavones acylated with methoxyhydroxycinnamic acids and p-coumaric acid derivatives, isoorientin 7-O-glucoside, other flavonoids | Water deficiency has increased chrysoeriol and apigenin glycoconjugates acylated with methoxylated hydroxycinnamic acids | (Piasecka, et al., 2017) |
| Corn |  |  |  |  |  |  |
| blue and red pigmented corn seeds | 80% MetOH | Untargeted | NMR | Polyphenolic compounds | Increased in the phenolic contents were directly correlated to an increase in spouting time, except when the temperature condition is at 10 °C. | (Carreño-Carrillo, Sánchez, Verduzco, & Herbert-Pucheta, 2021) |
| n=368 diverse inbred  lines | - | Untargeted | LC-MS/MS | 576 metabolites were identified including flavonoids (36 known), polyphenols, and phenolamide. | Flavonoids and phenolamides potentially played important roles in the domestications of maize and rice | (Deng, et al., 2020) |
| n= 368 diverse corn inbred lines | Absolute MetOH | Targeted | LC-MS/MS | 983 metabolites quantified:  49 metabolites identified included flavonoids, 28 flavones | The identified loci associated with flavonoids were direct targets of (or regulated by) locus *P1*. | (Wen, et al., 2014) |
| n=6 (including transgenic lines) | 50% MetOH | Untargeted | FT-ICR-MS | 145-1317 metabolites were identified depending on the solvent used. Includes phenolic compounds and flavonoids like apigeninidin. | FT-ICR-MS tentatively confirmed the presence of flavonoids like apigeninidin. The metabolic pathways are altered in transgenic lines. | (Leon, et al., 2009) |
| Oat |  |  |  |  |  |  |
| n=1 | 75% methanol 24.9% water 0.1%  formic acid | Untargeted | UHPLC-PDA-MS | Metabolites include phenolics and avenanthramide | The phenolic compounds were up-regulated in response to increased nitrogen supplementation are caffeic acid,  caffeoyl putrescine and some of sinapoyl glucose isomers. | (Allwood, et al., 2019) |
| n=1 *var.* Sang | 85% MetOH and diethyl ether | Targeted | GC-MS | 89 metabolites were identified, 32 of which are were trimethylsilyl derivatives of phenolic acids, their esters and aldehydes | Oat together with rye showed higher concentration of the most abundant phenolics acids compared to wheat and barley. | (Khakimov, Bak, & Engelsen, 2014) |
| Rice |  |  |  |  |  |  |
| n=394 | Methyl tert-butyl ether:MetOH (1:4) | Untargeted | GC-TOF-MS | 117 metabolites identified including phenolics and flavonoids | The red rice belonging to haplotype 4 has shown accumulation of catechin content and reduced glycemic index. | (Brotman, et al., 2021) |
| n=2 (red (cv. *TamTam*) and black (cv. *Artemide*) | 1.2-propanediol + water or lactic acid | Targeted | UPLC-MS/MS | 89 phenolic compounds were detected belonging to flavonoids (52%), phenolic acids (33%), other polyphenols (8%), lignans (6%) and stilbenes (1%) classes | Acidic deep eutectic extraction solvents demonstrated greater concentration of phenolic compounds in the extracts. Regardless of the solvents, the antioxidant capacity of black rice is higher than that of red rice. | (Santos, et al., 2021) |
| n=5 (including Chinese wild rice) | Absolute MetOH | Untargeted | UHPLC-QqQ-MS | A total of 159 flavonoids were identified. | Candidate genes were identified in the flavonoid biosynthetic pathway. | (Yu, et al., 2021) |
| n=16 | methanol/water  /chloroform (2.5:1:1) | Untargeted | GC×GC-TOF-MS, HS-SPME-GC-TOF-MS, GC-qMS, GC-FID, HPLC-MS, and HPLC-UV | 110 metabolites including phenolic acids, flavonoids, anthocyanins, etc. | Pathway analysis has revealed that black rice was abundant in secondary metabolites, however, has relatively low levels of primary metabolites compared with red rice. | (T. J. Kim, et al., 2021) |
| n=3 | 70% EtOH | Targeted | GC-MS | 46 metabolites were identified including protocatechuic acid | Vanillic acid and protocatechuic acid can distinguish the black and red rice samples. | (Kotamreddy, Hansda, & Mitra, 2020) |
| n=6 (Chinese and North American widl rice) | 70% MetOH | Untargeted | UHPLC-QqQ-MS | 672 metabolites were identified in which 124 phenolic acids and flavonoids included 5 anthocyanins, 7 benzoic acid derivatives, 4 catechin derivatives, 7 coumarins, 8 flavanones, 30 flavones, 19 flavone C-glycosides, 18 flavonol, 4 flavonolignans, 18 hydroxycinnamoyl derivatives, 1 isoflavone, and 3 proanthocyanidins | Out of 672, 357 metabolites showed differential expression (160 up-regulated versus 197 down-regulated). There is an enrichment in the phenylpropanoid biosynthesis pathway in the wild rice. | (Yan, et al., 2019) |
| n=3 | 70% or 100% EtOH | Untargeted | ^1^H-NMR | 34 metabolites were detected including phenolic acids such as sinapic acid, ferulic acid, p-hydroxybenzoic acid, gallic acid, and vannilic acid. | Higher phenolic compounds were extracted from using 100% EtOH. | (Pramai, et al., 2018) |
| n=5 black rice | methanol/water  /chloroform (2.5:1:1) | Untargeted | GC-TOF-MS | A total of 52 metabolites were identified, including 7 phenolic acids. | Hierarchical clustering analysis demonstrated positive relationship between all phenolic and shikimic acids. | (J. K. Kim, et al., 2013) |
| n=10 | ice-cold methanol/water (4∶1) | Untargeted | UPLC-MS | A total of 3,097 compounds were detected | Phenolics constitutes nonsynonymous SNPs and SNPs in the 5′ and 3′ untranslated regions for genes in their respective biosynthesis pathways. | (Heuberger, et al., 2010) |
| Rye |  |  |  |  |  |  |
| n=2 rye flour | MeOH:H2O:  HCOOH ratio of 80:19.9:0.1 | Untargeted | LC–QTOF-MS/MS | 110 metabolites were significantly identified in sourdough rye, including phenolic acids, flavonoids, and lignans. | Three microbial metabolites of phenolic acids such as dihydroferulic acid, dihydrocaffeic acid and dihydrosinapic acid increased in sourdough fermentation. | (Koistinen, et al., 2018) |
| n=1 (*var.* Petkus) | 85% MetOH and diethyl ether | Targeted | GC-MS | 89 metabolites were identified, including phenolics | Cinnamic acid derived phenolics  such as ferulic, sinapinic and syringic acids are abundant in rye. | (Khakimov, et al., 2014) |
| - | 75% MetOH and 0.1% formic acid | Untargeted | UPLC-qTOF-MS | 10 oligomeric  sesqui- and dilignans were identified in rye bran. | New lignans were detected such as buddlenol- and hedyotisol-type lignans. | (Hanhineva, et al., 2012) |
| Sorghum |  |  |  |  |  |  |
| n=3 (red, white, and black) | 70% MetOH | Targeted | LC-ESI-MS/MS | 651 metabolites identified phenolics, flavonoids, and anthocyanins. | Cyanidin O-malonyl-malonyl hexoside, cyanidin O-acetylhexoside, and cyanidin 3-O-glucosyl-malonylglucoside were significantly upregulated in red sorghum seeds. | (Zhou, et al., 2020) |
| n=5 | 1% formic acid in 80% MetOH | Untargeted | UHPLC-ESI/QTOF-MS | Around 36 compounds belonging to anthocyanins, dihydroflavanols, flavonols, flavanones, and lignans were selected using OPLS-DA model in relation to *in vitro* starch digestion. | Polyphenols have modulated the *in vitro* starch digestibility after cooking sorghum. | (Rocchetti, et al., 2020) |
| n=3 | 80% MetOH | Untargeted | LC-ESIQTOF-MS | 72 discriminating metabolites including phenolic compounds | 3-deoxyanthocynidin phytoalexins, apigeninidin, luteolinidin, and related conjugates served as antifungal compounds in sorghum. | (Tugizimana, Djami-Tchatchou, Steenkamp, Piater, & Dubery, 2019) |
| Wheat |  |  |  |  |  |  |
| n=8 soft winter wheat  cultivars | 80% EtOH | Untargeted and Targeted | UHPLC/ESI-QTOFMS | 248 metabolites were annotated comprising of hydroquinones, hydroxycinnamic acid amides, flavonoids, benzoxazinoids, lignans and other phenolics | The semi-polar fraction of the wheat samples were identified and quantified with 41 flavonoids, 60 hydroxycinnamic acid-conjugated biogenic amines, and 20 lignans. | (Tais, Schulz, & Böttcher, 2021) |
| n=2 | acetone-methanol-water (7:7:6) | Untargeted | UHPLC- HRAM/MS | Metabolites identified include phenolics compounds like phenolic acids, glycosylated apigenin and other flavonoids | Elevated CO_2_ has altered the phenolic concentration of the genetic lines. | (Geng, et al., 2020) |
| n=7 | 80% EtOH & ethyl acetate | Untargeted | UPLC-ESI-MS | 237 phenolic compounds were identified and further divided into 5 classes: flavonoids (85), phenolic acids (77), other polyphenols (51), lignans (16) and stilbenes (8) | The highest content of free and bound phenolics were determined in the free and bound fractions. | (Santos, et al., 2019) |
| n=3 ancient wheat cultivars | MetOH/  dichloromethane (50:50) | Untargeted | UHPLC-HRMS | 15 metabolites were identified including phenolic compounds. | Among the metabolites identified, alkylresorcinols, particularly the C21:0 and C19:0 homologues, discriminated the cultivars. | (Righetti, et al., 2016) |

Allwood, J. W., Xu, Y., Martinez-Martin, P., Palau, R., Cowan, A., Goodacre, R., Marshall, A., Stewart, D., & Howarth, C. (2019). Rapid UHPLC-MS metabolite profiling and phenotypic assays reveal genotypic impacts of nitrogen supplementation in oats. *Metabolomics, 15*, 1-19.

Andersson, A. A., Lampi, A.-M., Nystrom, L., Piironen, V., Li, L., Ward, J. L., Gebruers, K., Courtin, C. M., Delcour, J. A., & Boros, D. (2008). Phytochemical and dietary fiber components in barley varieties in the HEALTHGRAIN diversity screen. *Journal of agricultural and food chemistry, 56*, 9767-9776.

Andreasen, M. F., Christensen, L. P., Meyer, A. S., & Hansen, Å. (2000). Content of phenolic acids and ferulic acid dehydrodimers in 17 Rye (Secale c ereale L.) Varieties. *Journal of agricultural and food chemistry, 48*, 2837-2842.

Bei, Q., Chen, G., Liu, Y., Zhang, Y., & Wu, Z. (2018). Improving phenolic compositions and bioactivity of oats by enzymatic hydrolysis and microbial fermentation. *Journal of Functional Foods, 47*, 512-520.

Bei, Q., Liu, Y., Wang, L., Chen, G., & Wu, Z. (2017). Improving free, conjugated, and bound phenolic fractions in fermented oats (Avena sativa L.) with Monascus anka and their antioxidant activity. *Journal of Functional Foods, 32*, 185-194.

Boukid, F., Dall’Asta, M., Bresciani, L., Mena, P., Del Rio, D., Calani, L., Sayar, R., Seo, Y. W., Yacoubi, I., & Mejri, M. (2019). Phenolic profile and antioxidant capacity of landraces, old and modern Tunisian durum wheat. *European Food Research and Technology, 245*, 73-82.

Brotman, Y., Llorente‐Wiegand, C., Oyong, G., Badoni, S., Misra, G., Anacleto, R., Parween, S., Pasion, E., Tiozon Jr, R. N., & Anonuevo, J. J. (2021). The genetics underlying metabolic signatures in a brown rice diversity panel and their vital role in human nutrition. *The Plant Journal, 106*, 507-525.

Carreño-Carrillo, C. V., Sánchez, E. V., Verduzco, C. V., & Herbert-Pucheta, J. E. (2021). Polyphenol-based nuclear magnetic resonance non-targeted metabolomics of temperature-and time-controlled blue and red maize sprouting. *SN Applied Sciences, 3*, 1-10.

Carvalho, D. O., Curto, A. F., & Guido, L. F. (2015). Determination of phenolic content in different barley varieties and corresponding malts by liquid chromatography-diode array detection-electrospray ionization tandem mass spectrometry. *Antioxidants, 4*, 563-576.

Deng, M., Zhang, X., Luo, J., Liu, H., Wen, W., Luo, H., Yan, J., & Xiao, Y. (2020). Metabolomics analysis reveals differences in evolution between maize and rice. *The Plant Journal, 103*, 1710-1722.

Drawbridge, P. C., Apea-Bah, F., Hornung, P. S., & Beta, T. (2021). Bioaccessibility of phenolic acids in Canadian hulless barley varieties. *Food Chemistry, 358*, 129905.

Dykes, L., & Rooney, L. W. (2006). Sorghum and millet phenols and antioxidants. *Journal of Cereal Science, 44*, 236-251.

Ge, X., Jing, L., Zhao, K., Su, C., Zhang, B., Zhang, Q., Han, L., Yu, X., & Li, W. (2021). The phenolic compounds profile, quantitative analysis and antioxidant activity of four naked barley grains with different color. *Food Chemistry, 335*, 127655.

Geng, P., Sun, J., Chen, P., Li, Y., Peng, B., Harnly, J. M., & Bunce, J. (2020). A systematic approach to determine the impact of elevated CO2 levels on the chemical composition of wheat (Triticum aestivum). *Journal of Cereal Science, 95*, 103020.

Gotti, R., Amadesi, E., Fiori, J., Bosi, S., Bregola, V., Marotti, I., & Dinelli, G. (2018). Differentiation of modern and ancient varieties of common wheat by quantitative capillary electrophoretic profile of phenolic acids. *Journal of Chromatography A, 1532*, 208-215.

Han, Z., Ahsan, M., Adil, M. F., Chen, X., Nazir, M. M., Shamsi, I. H., Zeng, F., & Zhang, G. (2020). Identification of the gene network modules highly associated with the synthesis of phenolics compounds in barley by transcriptome and metabolome analysis. *Food Chemistry, 323*, 126862.

Hanhineva, K., Rogachev, I., Aura, A.-M., Aharoni, A., Poutanen, K., & Mykkänen, H. (2012). Identification of novel lignans in the whole grain rye bran by non-targeted LC–MS metabolite profiling. *Metabolomics, 8*, 399-409.

Heiniö, R.-L., Liukkonen, K.-H., Myllymäki, O., Pihlava, J.-M., Adlercreutz, H., Heinonen, S.-M., & Poutanen, K. (2008). Quantities of phenolic compounds and their impacts on the perceived flavour attributes of rye grain. *Journal of Cereal Science, 47*, 566-575.

Heuberger, A. L., Lewis, M. R., Chen, M.-H., Brick, M. A., Leach, J. E., & Ryan, E. P. (2010). Metabolomic and functional genomic analyses reveal varietal differences in bioactive compounds of cooked rice. *PloS one, 5*, e12915.

Hu, Q.-p., & Xu, J.-g. (2011). Profiles of carotenoids, anthocyanins, phenolics, and antioxidant activity of selected color waxy corn grains during maturation. *Journal of agricultural and food chemistry, 59*, 2026-2033.

Huang, S.-H., & Ng, L.-T. (2012). Quantification of polyphenolic content and bioactive constituents of some commercial rice varieties in Taiwan. *Journal of Food Composition and Analysis, 26*, 122-127.

Irakli, M. N., Samanidou, V. F., Biliaderis, C. G., & Papadoyannis, I. N. (2012). Simultaneous determination of phenolic acids and flavonoids in rice using solid‐phase extraction and RP‐HPLC with photodiode array detection. *Journal of Separation Science, 35*, 1603-1611.

Khakimov, B., Bak, S., & Engelsen, S. B. (2014). High-throughput cereal metabolomics: Current analytical technologies, challenges and perspectives. *Journal of Cereal Science, 59*, 393-418.

Kim, J. K., Park, S.-Y., Lim, S.-H., Yeo, Y., Cho, H. S., & Ha, S.-H. (2013). Comparative metabolic profiling of pigmented rice (Oryza sativa L.) cultivars reveals primary metabolites are correlated with secondary metabolites. *Journal of Cereal Science, 57*, 14-20.

Kim, T. J., Kim, S. Y., Park, Y. J., Lim, S.-H., Ha, S.-H., Park, S. U., Lee, B., & Kim, J. K. (2021). Metabolite Profiling Reveals Distinct Modulation of Complex Metabolic Networks in Non-Pigmented, Black, and Red Rice (Oryza sativa L.) Cultivars. *Metabolites, 11*, 367.

Koistinen, V. M., Mattila, O., Katina, K., Poutanen, K., Aura, A.-M., & Hanhineva, K. (2018). Metabolic profiling of sourdough fermented wheat and rye bread. *Scientific reports, 8*, 1-11.

Kotamreddy, J. N. R., Hansda, C., & Mitra, A. (2020). Semi-targeted metabolomic analysis provides the basis for enhanced antioxidant capacities in pigmented rice grains. *Journal of Food Measurement and Characterization*, 1-9.

Kulichová, K., Sokol, J., Nemeček, P., Maliarová, M., Maliar, T., Havrlentová, M., & Kraic, J. (2019). Phenolic compounds and biological activities of rye (Secale cereale L.) grains. *Open Chemistry, 17*, 988-999.

Leon, C., Rodriguez-Meizoso, I., Lucio, M., Garcia-Cañas, V., Ibañez, E., Schmitt-Kopplin, P., & Cifuentes, A. (2009). Metabolomics of transgenic maize combining Fourier transform-ion cyclotron resonance-mass spectrometry, capillary electrophoresis-mass spectrometry and pressurized liquid extraction. *Journal of Chromatography A, 1216*, 7314-7323.

Ma, D., Li, Y., Zhang, J., Wang, C., Qin, H., Ding, H., Xie, Y., & Guo, T. (2016). Accumulation of phenolic compounds and expression profiles of phenolic acid biosynthesis-related genes in developing grains of white, purple, and red wheat. *Frontiers in plant science, 7*, 528.

Méndez-Lagunas, L. L., Cruz-Gracida, M., Barriada-Bernal, L. G., & Rodríguez-Méndez, L. I. (2020). Profile of phenolic acids, antioxidant activity and total phenolic compounds during blue corn tortilla processing and its bioaccessibility. *Journal of food science and technology, 57*, 4688-4696.

Miafo, A.-P. T., Koubala, B. B., Kansci, G., & Muralikrishna, G. (2019). Free sugars and non-starch polysaccharides–phenolic acid complexes from bran, spent grain and sorghum seeds. *Journal of Cereal Science, 87*, 124-131.

Miafo, A. P. T., Koubala, B. B., Kansci, G., & Muralikrishna, G. (2020). Antioxidant properties of free and bound phenolic acids from bran, spent grain, and sorghum seeds. *Cereal Chemistry, 97*, 1236-1243.

Multari, S., Pihlava, J.-M., Ollennu-Chuasam, P., Hietaniemi, V., Yang, B., & Suomela, J.-P. (2018). Identification and quantification of avenanthramides and free and bound phenolic acids in eight cultivars of husked oat (Avena sativa L) from Finland. *Journal of agricultural and food chemistry, 66*, 2900-2908.

Pang, Y., Ahmed, S., Xu, Y., Beta, T., Zhu, Z., Shao, Y., & Bao, J. (2018). Bound phenolic compounds and antioxidant properties of whole grain and bran of white, red and black rice. *Food Chemistry, 240*, 212-221.

Piasecka, A., Sawikowska, A., Kuczyńska, A., Ogrodowicz, P., Mikołajczak, K., Krystkowiak, K., Gudyś, K., Guzy‐Wróbelska, J., Krajewski, P., & Kachlicki, P. (2017). Drought‐related secondary metabolites of barley (Hordeum vulgare L.) leaves and their metabolomic quantitative trait loci. *The Plant Journal, 89*, 898-913.

Pihlava, J.-M., Nordlund, E., Heiniö, R.-L., Hietaniemi, V., Lehtinen, P., & Poutanen, K. (2015). Phenolic compounds in wholegrain rye and its fractions. *Journal of Food Composition and Analysis, 38*, 89-97.

Pramai, P., Hamid, N. A. A., Mediani, A., Maulidiani, M., Abas, F., & Jiamyangyuen, S. (2018). Metabolite profiling, antioxidant, and α-glucosidase inhibitory activities of germinated rice: nuclear-magnetic-resonance-based metabolomics study. *Journal of food and drug analysis, 26*, 47-57.

Righetti, L., Rubert, J., Galaverna, G., Folloni, S., Ranieri, R., Stranska-Zachariasova, M., Hajslova, J., & Dall’Asta, C. (2016). Characterization and discrimination of ancient grains: A metabolomics approach. *International Journal of Molecular Sciences, 17*, 1217.

Rocchetti, G., Giuberti, G., Busconi, M., Marocco, A., Trevisan, M., & Lucini, L. (2020). Pigmented sorghum polyphenols as potential inhibitors of starch digestibility: An in vitro study combining starch digestion and untargeted metabolomics. *Food Chemistry, 312*, 126077.

Santos, M. C. B., Barouh, N., Durand, E., Baréa, B., Robert, M., Micard, V., Lullien-Pellerin, V., Villeneuve, P., Cameron, L. C., & Ryan, E. P. (2021). Metabolomics of Pigmented Rice Coproducts Applying Conventional or Deep Eutectic Extraction Solvents Reveal a Potential Antioxidant Source for Human Nutrition. *Metabolites, 11*, 110.

Santos, M. C. B., da Silva Lima, L. R., Nascimento, F. R., do Nascimento, T. P., Cameron, L. C., & Ferreira, M. S. L. (2019). Metabolomic approach for characterization of phenolic compounds in different wheat genotypes during grain development. *Food Research International, 124*, 118-128.

Shao, Y., Xu, F., Sun, X., Bao, J., & Beta, T. (2014). Phenolic acids, anthocyanins, and antioxidant capacity in rice (Oryza sativa L.) grains at four stages of development after flowering. *Food Chemistry, 143*, 90-96.

Soycan, G., Schär, M. Y., Kristek, A., Boberska, J., Alsharif, S. N., Corona, G., Shewry, P. R., & Spencer, J. P. (2019). Composition and content of phenolic acids and avenanthramides in commercial oat products: Are oats an important polyphenol source for consumers? *Food chemistry: X, 3*, 100047.

Suriano, S., Iannucci, A., Codianni, P., Fares, C., Russo, M., Pecchioni, N., Marciello, U., & Savino, M. (2018). Phenolic acids profile, nutritional and phytochemical compounds, antioxidant properties in colored barley grown in southern Italy. *Food Research International, 113*, 221-233.

Tais, L., Schulz, H., & Böttcher, C. (2021). Comprehensive profiling of semi‐polar phytochemicals in whole wheat grains (Triticum aestivum) using liquid chromatography coupled with electrospray ionization quadrupole time‐of‐flight mass spectrometry. *Metabolomics, 17*, 1-18.

Tian, W., Chen, G., Tilley, M., & Li, Y. (2021). Changes in phenolic profiles and antioxidant activities during the whole wheat bread-making process. *Food Chemistry, 345*, 128851.

Tugizimana, F., Djami-Tchatchou, A. T., Steenkamp, P. A., Piater, L. A., & Dubery, I. A. (2019). Metabolomic analysis of defense-related reprogramming in Sorghum bicolor in response to Colletotrichum sublineolum infection reveals a functional metabolic web of phenylpropanoid and flavonoid pathways. *Frontiers in plant science, 9*, 1840.

Vichapong, J., Sookserm, M., Srijesdaruk, V., Swatsitang, P., & Srijaranai, S. (2010). High performance liquid chromatographic analysis of phenolic compounds and their antioxidant activities in rice varieties. *LWT-Food Science and Technology, 43*, 1325-1330.

Wen, W., Li, D., Li, X., Gao, Y., Li, W., Li, H., Liu, J., Liu, H., Chen, W., & Luo, J. (2014). Metabolome-based genome-wide association study of maize kernel leads to novel biochemical insights. *Nature communications, 5*, 1-10.

Yan, N., Du, Y., Liu, X., Chu, M., Shi, J., Zhang, H., Liu, Y., & Zhang, Z. (2019). A comparative UHPLC-QqQ-MS-based metabolomics approach for evaluating Chinese and North American wild rice. *Food Chemistry, 275*, 618-627.

Yang, T., Guang Hu, J., Yu, Y., Li, G., Guo, X., Li, T., & Liu, R. H. (2019). Comparison of phenolics, flavonoids, and cellular antioxidant activities in ear sections of sweet corn (Zea mays L. saccharata Sturt). *Journal of Food Processing and Preservation, 43*, e13855.

Yu, X., Yang, T., Qi, Q., Du, Y., Shi, J., Liu, X., Liu, Y., Zhang, H., Zhang, Z., & Yan, N. (2021). Comparison of the contents of phenolic compounds including flavonoids and antioxidant activity of rice (Oryza sativa) and Chinese wild rice (Zizania latifolia). *Food Chemistry, 344*, 128600.

Zeng, Z., Liu, C., Luo, S., Chen, J., & Gong, E. (2016). The profile and bioaccessibility of phenolic compounds in cereals influenced by improved extrusion cooking treatment. *PloS one, 11*, e0161086.

Zhang, Y., Wang, L., Yao, Y., Yan, J., & He, Z. (2012). Phenolic acid profiles of Chinese wheat cultivars. *Journal of Cereal Science, 56*, 629-635.

Zhou, M., Malhan, N., Ahkami, A. H., Engbrecht, K., Myers, G., Dahlberg, J., Hollingsworth, J., Sievert, J. A., Hutmacher, R., & Madera, M. (2020). Top-down mass spectrometry of histone modifications in sorghum reveals potential epigenetic markers for drought acclimation. *Methods, 184*, 29-39.

Zhu, Y., Li, T., Fu, X., Abbasi, A. M., Zheng, B., & Liu, R. H. (2015). Phenolics content, antioxidant and antiproliferative activities of dehulled highland barley (Hordeum vulgare L.). *Journal of Functional Foods, 19*, 439-450.
